# The Ubiquitin-Proteasome System Facilitates Membrane Fusion and Uncoating during Coronavirus Entry

**DOI:** 10.3390/v15102001

**Published:** 2023-09-26

**Authors:** Xiao Yuan, Xiaoman Zhang, Huan Wang, Xiang Mao, Yingjie Sun, Lei Tan, Cuiping Song, Xusheng Qiu, Chan Ding, Ying Liao

**Affiliations:** 1 Department of Avian Diseases, Shanghai Veterinary Research Institute, Chinese Academy of Agricultural Sciences, Shanghai 200241, China; 13331872602@163.com (X.Y.); wxz42609@163.com (X.Z.); wanghuan9292@163.com (H.W.); asters_m@163.com (X.M.); sunyingjie@shvri.ac.cn (Y.S.); tanlei@shvri.ac.cn (L.T.); scp@shvri.ac.cn (C.S.); xsqiu1981@shvri.ac.cn (X.Q.); shoveldeen@shvri.ac.cn (C.D.); 2 Jiangsu Co-Innovation Center for Prevention and Control of Important Animal Infectious Diseases and Zoonoses, Yangzhou 225009, China

**Keywords:** ubiquitin proteasome system, ubiquitylation, virus entry, membrane fusion, uncoating

## Abstract

Although the involvement of the ubiquitin-proteasome system (UPS) in several coronavirus-productive infections has been reported, whether the UPS is required for infectious bronchitis virus (IBV) and porcine epidemic diarrhea virus (PEDV) infections is unclear. In this study, the role of UPS in the IBV and PEDV life cycles was investigated. When the UPS was suppressed by pharmacological inhibition at the early infection stage, IBV and PEDV infectivity were severely impaired. Further study showed that inhibition of UPS did not change the internalization of virus particles; however, by using R18 and DiOC-labeled virus particles, we found that inhibition of UPS prevented the IBV and PEDV membrane fusion with late endosomes or lysosomes. In addition, proteasome inhibitors blocked the degradation of the incoming viral protein N, suggesting the uncoating process and genomic RNA release were suppressed. Subsequently, the initial translation of genomic RNA was blocked. Thus, UPS may target the virus-cellular membrane fusion to facilitate the release of incoming viruses from late endosomes or lysosomes, subsequently blocking the following virus uncoating, initial translation, and replication events. Similar to the observation of proteasome inhibitors, ubiquitin-activating enzyme E1 inhibitor PYR-41 also impaired the entry of IBV, enhanced the accumulation of ubiquitinated proteins, and depleted mono-ubiquitin. In all, this study reveals an important role of UPS in coronavirus entry by preventing membrane fusion and identifies UPS as a potential target for developing antiviral therapies for coronavirus.

## 1. Introduction

Coronaviruses are enveloped, plus-strand RNA viruses belonging to the family *Coronaviridae*, which are common pathogens in many animal species. This virus family harbors the longest genome among RNA viruses, with a size of approximately 25 to 32 kilobases (kb). In most cases, coronaviruses cause respiratory and/or intestinal tract diseases. Several highly pathogenic coronaviruses infect humans and cause pandemics, such as Severe Acute Respiratory Syndrome Coronavirus 2 (SARS-CoV-2), SARS-CoV, and the Middle East Respiratory Syndrome Virus (MERS-CoV); meanwhile, many coronaviruses circulate in animals and cause economic loss in livestock, poultry, and pets [1,2]. IBV was the first identified avian coronavirus in the 1930s and belongs to the genus Gamma *coronavirus*. It is one of the dominant pathogens causing highly contagious infectious bronchitis, circulating in poultry farms for nearly a century, and leading to significant economic loss [3]. Porcine epidemic diarrhea virus (PEDV) belongs to the genus *alpha coronavirus* and was first discovered in England in the 1970s [4]. The highly virulent PEDV has become increasingly problematic in Asian countries [5,6,7,8] and also reemerged in America in 2013 [9]. PEDV infects the small intestine and causes diarrhea, with up to 95% mortality in suckling piglets [10].

The coronavirus genome encodes 2 polyproteins 1a and 1ab, four structural proteins, namely, spike protein (S), membrane protein (M), small envelope protein (E), nucleocapsid protein (N), and accessory proteins [1,2,11,12]. The coronavirus infection starts with the attachment of the virus to specific cellular receptors, which is mediated by the S protein [13]. After endocytosis, both IBV and PEDV traffic along the endocytic pathway for a successful infection [14,15,16]. The membrane fusion occurs within late endosomes and lysosomes after the S protein is cleaved by a low-pH-dependent protease; after that, the nucleocapsid is released into the cytosol, and the viral genomic RNA is uncoated. The first round of viral protein translation is initiated by using the positive-stranded genomic RNA as a template, producing polyproteins 1a and 1ab; the latter is translated by a ribosomal frameshift mechanism [17,18]. The two polyproteins are then processed by two internal proteases, named papain-like protease and 3C-like protease, yielding 15–16 non-structural proteins (nsp1–nsp16) [19,20]. Among them, nsp3, nsp4, and nsp6 harbor hydrophobic transmembrane domains and are responsible for the formation of double membrane vesicles (DMVs), which accommodate the viral replication-transcription complex (TRS). The TRS is comprised of a set of replicases: nsp7 and nsp8 (primer synthase), nsp9, nsp10, nsp12 (RNA-dependent RNA polymerase), nsp13 (helicase), nsp14 (exoribonuclease), nsp15 (endoribonuclease), and nsp16 (2′-O-methyltransferase) [21,22]. The RTC synthesizes virus genomic RNA and a nested set of sub-genomic mRNAs, which are then translated into viral proteins [23]. Together with the newly synthesized genomic RNA, the structural proteins are assembled into progeny virions, which bud through the membranes of the ER to the Golgi intermediate compartment (ERGIC) [17,24]. The newly produced virions are subsequently released by exocytosis [25,26].

In eukaryotic cells, the ubiquitin-proteasome system (UPS) is the major intracellular pathway for functional modification and degradation of cellular proteins. It plays a key role in the regulation of many fundamental cellular processes, involving apoptosis, cell cycle regulation, signal transduction, antigen processing, and transcriptional regulation [27,28,29]. Ubiquitylation is involved in conjugating the 76 amino acid ubiquitin on the lysine residues of target proteins [30,31], processed by an enzymatic cascade: the E1 ubiquitin-activating enzyme presents an ubiquitin to the E2 ubiquitin-conjugating enzyme, then the E3 ubiquitin-ligase transfers the ubiquitin from the E2 enzyme to the protein substrates [32,33]. Ubiquitylation is commonly associated with proteasomal protein degradation or protein conformational/functional change [34,35]. Degradation of intracellular proteins and misfolded polypeptides tagged with polyubiquitin chains is a highly complex, tightly regulated process that is carried out by the 26S proteasomes [30,36,37,38,39,40].

All viruses exploit and manipulate the infrastructure and metabolism of their host cells for their own advantage. It is not surprising that UPS has also been implicated in the virus life cycle and virus-host interplay [41,42,43]. It has been reported that UPS plays an important role in infection by a variety of viruses [44]. On one hand, UPS is utilized by viruses to maintain the proper function and level of viral proteins [45]. E3 ligase RNF5 also mediates the ubiquitination of SARS-CoV-2 M protein at K15 to enhance the interaction of M and E proteins, which ensures the uniform size of viral particles for viral maturation and mediates virion release by using autophagosomes [46]. Zika virus envelope protein is ubiquitinated to facilitate extracellular interactions with receptors, thereby driving virus entry and pathogenesis [47]. Some viral proteins are degraded by UPS to maintain the proper ratio among viral proteins, which is critical for productive viral infection and/or evade recognition by the host immune system. The UPS also assists in several steps of the initiation of infection, including the endosomal escape of the entering virions, intracellular transport of incoming nucleocapsids, and uncoating of the viral genome, which were found in murine coronavirus, Japanese encephalitis virus (JEV), and dengue virus [48,49,50,51,52]. On the other hand, UPS constitutes a host defense mechanism to eliminate viral proteins [53]. For instance, SARS-CoV and SARS-CoV-2 structural protein E was ubiquitinated by E3 ligase RNF5 and degraded by UPS [54,55]; West Nile Virus (WNV) capsid protein is ubiquitinated by the cellular E3 ligase, MKRN1, followed by proteasomal degradation; overexpression of MKRN1 significantly reduced WNV proliferation in 293T cells [56,57]. To combat the host anti-viral machinery, viruses also employ UPS to degrade or inactivate cellular proteins, which limit viral growth [58,59]. It has been reported that HIV hijacks the UPS to mediate defense against several cellular restriction factors [30,60]. Many virus infections are sensitive to proteasome inhibitors, including coronavirus [51,52,61,62], herpesvirus [63], porcine circovirus [64], influenza A virus [65], HIV [30], human astrovirus [66], hepatitis B virus [67], dengue virus [50], and JEV [49]. Thus, analyzing the role of UPS in the process of viral infection is helpful to understand the importance of host-related systems in viral infection and design anti-viral drugs.

Previous studies have reported that UPS plays an important role during various stages of the murine coronavirus infection cycle [51,52]. It has been shown that the UPS facilitates the transfer of murine coronavirus from the endosome to the cytoplasm during virus entry [52]. However, whether UPS blocks pan-coronavirus entry is unclear. In this study, we employed the *α coronavirus* PEDV and *γ coronavirus* IBV to investigate the role of the UPS on the coronavirus life cycle by using proteasome chemical inhibitors MG132, epoxomicin, Bortezomib, and ubiquitin-activating enzyme E1 inhibitor PYR-41. By using these inhibitors, we found that UPS facilitates IBV and PEDV membrane fusion with late endosomes or lysosomes and subsequent uncoating/initial translation.

## 2. Materials and Methods

### 2.1. Cells and Viruses

Vero cells (African green monkey kidney epithelial cells) (ATCC^®^CCL-81™) and DF-1 cells (chicken embryo fibroblasts) (ATCC^®^ CRL-12203™) were purchased from ATCC (American Type Culture Collection, Manassas, VA, USA) and maintained in Dulbecco’s modified Eagle medium (DMEM) (Gibco™, Thermo Fisher Scientific, Waltham, MA, USA) with 4500 mg/L glucose, supplemented with 10% fetal bovine serum (FBS) (Hyclone, South Logan, UT, USA), 100 units/mL penicillin, and 100 μg/mL streptomycin (Invitrogen, Carlsbad, CA, USA).

The Beaudette strain of IBV (ATCC VR-22) adapted to Vero cells is a gift from Prof. Liu Dingxiang (Huanan Agricultural University, Guangzhou, China). PEDV (HLJBY strain) is kindly provided by Prof. Mao Xiang.

### 2.2. Chemicals and Antibodies

The UPS inhibitors MG132 (S2619), Epoxomicin (S7038), and Bortezomib (S1013) and the Ubiquitin-activating enzyme E1 inhibitor PYR-41 (S7129) were purchased from Selleckchem (Houston, TX, USA) [43,64,65,68].

Anti-IBV N and anti-IBV nsp3 polyclonal antibodies were obtained through immunization of rabbits with respective antigens. Anti-PEDV N is a gift from Prof. Zhou Yanjun (Shanghai Veterinary Research Institute, Shanghai, China). Anti-β-actin (A1978) was purchased from Sigma-Aldrich (St. Louis, MO, USA). Anti-ubiquitin (#3933), anti-Rab5 (#3547s), anti-Rab7 (#9367s), and anti-LAMP1 (#9091s), fluorescein isothiocyanate (FITC)-conjugated anti-mouse and anti-rabbit immunoglobulin G (IgG), and horseradish peroxidase (HRP)-conjugated anti-mouse and anti-rabbit IgG were purchased from Cell Signaling Technology@ (Danvers, MA, USA).

### 2.3. Cell Viability Assay

The viability of drug-treated cells was measured using the WST-1 cell proliferation and cytotoxicity assay kit (C0035, Beyotime, Haimen, China) according to the manufacturer’s instructions. Briefly, cells were seeded in a 96-well plate and treated with the indicated drug (MG132, Epoxomicin, or Bortezomib) for 12 hours. A total of 10 μL of WST-1 was added to each well and incubated for 1 h. The absorbance at 450 nm was monitored, and the reference wavelength was set at 630 nm.

### 2.4. Virus Infection and Drug Treatment

To test the effect of various UPS inhibitors on IBV infection, Vero cells and DF-1 cells were seeded into 6-well plates and cultured overnight. Cells were inoculated with IBV at MOI = 1 in serum-free medium and incubated at 4 °C for 1 h. The unbound virions were washed away with PBS, and the cells were replenished with serum-free culture medium and incubated at 37 °C. The proteasome inhibitors or ubiquitin-activating enzyme E1 inhibitor PYR-41 were added at the indicated time points. The cells were collected and subjected to western blot or SYBR green real-time RT-qPCR, and the culture medium was collected for the TCID_50_ assay.

### 2.5. Virus Internalization Assay

Vero or DF-1 cells in 6-well plates were incubated with IBV (MOI = 1) at 4 °C for 1 h. The unbound virions were washed away with PBS, and the cells were incubated at 37 °C in the presence of proteasome inhibitors. At 2 h.p.i., cells were treated with 1 mg/mL proteinase K (Invitrogen) for 15 min to remove the cell surface adsorbed but not internalized virus. Proteinase K was inactivated with 2 mM phenylmethylsulfonyl fluoride (PMSF) in PBS with 3% bovine serum albumin. Cells were then washed three times with PBS and subjected to RNA isolation and real-time RT-qPCR.

### 2.6. RNA Preparation and Real Time RT-qPCR

The cells were lysed with TRIZOL reagent (Invitrogen, Thermo Fisher Scientific, Carlsbad, CA, USA). One fifth volume of chloroform was added and mixed well with the cell lysates. The mixture was centrifuged at 10,000 × *g* for 15 min at 4 °C, and the supernatant was then mixed with an equal volume of 100% isopropanol and incubated at 4 °C for more than 30 min. RNA was precipitated by isopropanol and pelleted by centrifugation at 10,000× *g* for 20 min at 4 °C. The RNA pellets were washed twice with 70% RNase-free ethanol and dissolved in 30 μL of RNase-free water.

An amount of 2 µg of total RNA was used to perform reverse transcription using Expand reverse transcriptase (Roche, Basel, Switzerland) and oligo-dT/specific primers. An equal volume of cDNAs was then PCR-amplified using the SYBR green PCR master kit (Dongsheng Biotech, Guangdong, China). The specific primer sequences targeting IBV positive-stranded genomic RNA and negative intermediate genomic RNA were: 5′-TTTAGCAGAACATTTTGACGCAGAT-3′ and 5′-TTAGTAGAACCAACAAACAC GACAG-3′ [69].

### 2.7. Western Blot Analysis

Cells were lysed with 1× SDS loading buffer in the presence of 100 mM dithiothreitol and denatured at 100 °C for 5 min. Equivalent amounts of protein were separated by SDS-PAGE, followed by transfer onto polyvinylidene difluoride (PVDF) membranes (Bio-Rad Laboratories, Hercules, CA, USA) by electroblotting. Membranes were incubated with blocking buffer (5% fat-free milk in TBST) for 1 h, followed by incubation with appropriate antibodies (diluted in 5% BSA TBST) for another 1 h at room temperature. After washing three times with TBST, membranes were incubated with HRP-conjugated secondary antibody for 1 h and washed with TBST three times. Blots were developed with an enhanced chemiluminescence (ECL) detection system (GE Healthcare Life Sciences, Little Chalfont, Buckinghamshire, USA) and exposed to a Chemiluminescence gel imaging system (Tanon 5200, Shanghai, China).

### 2.8. Virus Titration

The viral titers were determined by a 50% infectious dose (TCID_50_) assay. A total of 10-fold serially diluted aliquots of IBV were applied to DF-1 cells in 96-well plates. After 1 h of adsorption, unbound viruses were removed, and the cells were washed by PBS and replaced with fresh DMEM. The plates were incubated at 37 °C, and the cytopathic effect (CPE) was observed after 3 days. The tissue culture TCID_50_ is calculated using Reed and Muench mathematical analysis [70].

### 2.9. R18 Labeling and R18/DIOC Labeling of Virus

IBV or PEDV particles were purified and concentrated as follows: Vero cells were infected with the virus for 24 h. The cell supernatant was centrifuged at 10,000× *g* for 15 min to remove cell debris and nuclei (JA-25.50 rotor, Beckman ultracentrifuge) (Beckman Coulter, Miami, FL, USA). The supernatants were centrifuged at 5000× *g* for 30 min through Amicon^®^ Ultra-15 Centrifugal Filter Devices (10-kDa cutoff) (Merck Millipore, Billerica, MA, USA), which provide fast ultrafiltration. For the R18 labeling, 100 μL of purified virus was incubated with 2.5 μL of 1.7 mM R18 (Molecular Probes, Eugne, OR, USA) on a rotary shaker for 1 h at room temperature [71]. To remove unincorporated dye, the virus was filtered through a 0.45 mm syringe filter (Millipore, Billerica, MA, USA) and used freshly for the assay. For the R18/DIOC labeling, 100 μL of purified virus was resuspended in 200 µL of phosphate-buffered saline (PBS) before incubation with a 3.3 mM DIOC and 6.7 mM R18 mixture (Molecular Probes, Eugene, OR, USA) [72]. Labeling was performed for 1 h at room temperature with gentle shaking. After finishing the labeling, the virus and dye mixture was re-suspended in 8 mL phosphate-buffered saline (PBS), and the excess unincorporated dye was removed with an Amicon^®^ Ultra-15 Centrifugal Filter Devices (10-kDa cutoff) (Merck Millipore, Billerica, MA, USA) by centrifugating for 60 min. The labeled virus was used freshly for the membrane fusion assay.

Cells were seeded on 4-well chamber slides and infected with R18-IBV, R18-DIOC-IBV, or R18-DIOC-PEDV at a MOI of 5. At indicated time points, cells were fixed with 4% paraformaldehyde for 10 min, washed three times with PBS, permeabilized with 0.2% Triton X-100 (Thermo Fisher Scientific, Carlsbad, CA, USA) for 10 min, and washed three times with PBS. Cells were then incubated with anti-Rab5, anti-Rab7, anti-LAMP1, anti-phalloidin (1:200 diluted in PBS, 5% BSA), or CTB (5 µg/mL), for 2 h, washed thrice with PBS, and then incubated with secondary antibody conjugated with FITC (DAKO, Glostrup, Denmark) for 2 h (1:200 diluted in PBS, 5% BSA), followed by PBS washing. Cells were next incubated with 0.1 µg/mL DAPI for 10 min and rinsed with PBS. Finally, the specimen was mounted with glass cover slips using fluorescent mounting medium (DAKO, Glostrup, Denmark) containing 15 mMNaN3. Images were collected with a LSM880 confocal laser-scanning microscope (Zeiss, Oberkochen, German).

### 2.10. Statistical Analysis

All data are presented as means ± standard deviations (SD), as indicated. A student’s *t*-test was used to compare data from pairs of treated or untreated groups. Statistical significance is indicated in the figure legends. All statistical analyses and calculations were performed using Graph Pad Prism 5 (Graph Pad Software Inc., La Jolla, CA, USA).

### 2.11. Densitometry

The intensities of corresponding bands were quantified using the Image J program (V.1.8.0, NIH) according to the manufacturer’s instructions.

## 3. Results

### 3.1. Proteasome Inhibitors Interfere with IBV Infection

**Figure 1:**
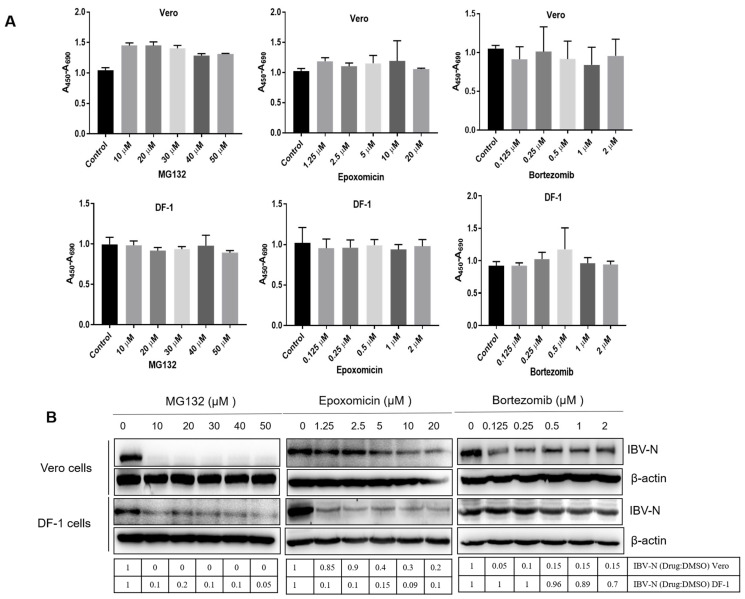
IBV infection is suppressed by treatment with proteasome inhibitors. (**A**) Vero cells and DF-1 cells were incubated with increasing concentrations of MG132 (0–50 μM), Epoxomicin (0–20 μM), or Bortezomib (0–2 μM) for 12 h and subjected to a cell viability assay using the WST-1 assay kit. The untreated cells were included as a control group. In total 450 nm is the determination wavelength, and 690 nm is the reference wavelength. The value of each sample was normalized to the control group and presented in a bar graph panel. Error bars represent the standard deviations from three replicates. (**B**) Vero and DF-1 cells were infected with 1 MOI of IBV for 1 h and incubated with increasing concentrations of MG132 (0–50 μM), Epoxomicin (0–20 μM), or Bortezomib (0–2 μM). To determine the virus infectivity, the expression of the IBV N protein was analyzed by western blot at 12 h.p.i. β-actin was measured as an internal loading control. The intensity of the IBV-N band was determined with image J, normalized to β-actin, and shown as a fold change of Drug:DMSO.

To investigate the role of UPR on the IBV life cycle, we first analyzed the effect of proteasome inhibition on IBV infection. We employed several inhibitors to suppress the proteasome-mediated protein degradation in IBV Beaudette strain-infected cells: MG132, a reversible and cell-permeable proteasome inhibitor [73], Epoxomicin, an irreversible proteasome inhibitor targeting the 20S subunit [74], and the 26S subunit proteasome inhibitor Bortezomib [75]. The cytotoxicity of these chemicals was determined. The IBV Beaudette strain permissive cell line Vero and chicken embryo fibroblast DF-1 were treated with working concentrations of MG132 (0–50 μM), Epoxomicin (0–20 μM), or Bortezomib (0–2 μM) for 12 h and subjected to cell viability assay by using the WST-1 assay kit [14]. The non-toxic concentration range of these inhibitors without an effect on cell viability is shown in Figure 1A. To determine whether inhibition of the proteasome interferes with IBV infection, Vero and DF-1 cells were infected with 1 MOI of IBV and incubated with increasing concentrations of these inhibitors. Vero cells are monkey cells permissive to IBV Beaudette and DF-1 cells are derived from the IBV host chicken. Both cell lines are susceptible to the IBV Beaudette infection. The expression of the IBV N protein was analyzed at 12 h.p.i., to determine the virus infectivity. As shown in Figure 1B, in both Vero and DF-1 cells, the expression of the IBV N protein was greatly decreased in cells treated with MG132 from 10 μM to 50 μM. The inhibition concentrations on IBV infectivity of Epoxomicin and Bortezomid in these two cell types were slightly different: Epoxomicin exerted a great inhibition effect on N protein level in DF-1 cells from 1.25 μM to 20 μM, while in Vero cells, a moderate inhibition effect was observed from 5 μM to 20 μM; the effect of Bortezomid on IBV replication was moderate in Vero cells, while there was no obvious inhibition effect in DF-1 cells. The above results suggest that the sensitivity to Epoxomicin and Bortezomid is slightly different in Vero and DF-1 cells. Furthermore, these inhibitors indeed exert a suppression effect on IBV infectivity, especially MG132 and Epoxomicin.

### 3.2. Proteasome Inhibitors Act at An Early Step of IBV Infection

**Figure 2:**
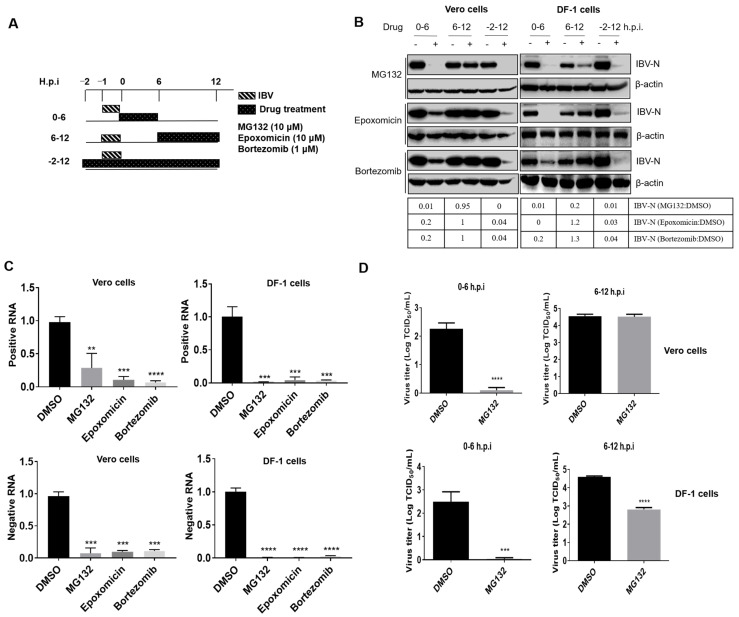
Proteasome inhibitors act in the early stages of IBV infection. (**A**) Experimental design for pulse treatment with different proteasome inhibitors. The cells were treated with inhibitors during different infection times and collected at 12 h.p.i. (**B**) Vero and DF-1 cells were infected with 1 MOI of IBV and incubated with MG132 (10 μM), Epoxomicin (10 μM), and Bortezomib (1 μM) at 0–6 h.p.i., 6–12 h.p.i., or −2–12 h.p.i. The cells were collected at 12 h.p.i., and IBV N protein was detected by western blot. β-actin was measured as an internal loading control. The intensity of the IBV-N band was determined with image J, normalized to β-actin, and shown as a fold change of MG132:DMSO, Epoxomicin:DMSO, and Bortezomib:DMSO. (**C**) Vero and DF-1 cells were infected with 1 MOI of IBV and incubated with MG132 (10 μM), Epoxomicin (10 μM), and Bortezomib (1 μM) at 0–6 h.p.i. The cells were collected at 12 h.p.i. for detection of positive and negative sense viral RNA levels by using RT-qPCR. (**D**) Vero and DF-1 cells were infected with 1 MOI of IBV and incubated with MG132 (10 μM) at 0–6 or 6–12 h.p.i. The culture medium was collected at 12 h.p.i. for detection of virus yield by using TCID_50_. The experiments in C and D were performed in triplicate, and the average values with stand errors were calculated and presented in a bar graph panel. The asterisk (*) represents the *p*-value of the statistical test. ** *p*-value < 0.05 (significant); *** *p*-value < 0.01 (very significant); **** *p*-value < 0.001 (highly significant).

In order to investigate whether proteasome inhibitors specifically block virus entry or subsequent events at later stages of the infection cycle, Vero and DF-1 cells were inoculated with IBV and treated with MG132 (10 μM), Epoxomicin (10 μM), or Bortezomib (1 μM) at several time points post-infection (0–6 h.p.i., 6–12 h.p.i., and −2–12 h.p.i.). The experimental setup scheme is shown in Figure 2A. Virus infectivity was examined at 12 h.p.i. As shown in Figure 2B, in both cell types, the presence of proteasome inhibitors at 0–6 h.p.i. and −2–12 h.p.i. greatly reduced the N protein expression level; however, when the proteasome activity was inhibited by chemicals at 6–12 h.p.i., the inhibition effect on IBV infectivity was not obvious, except for MG132 treatment in DF-1 cells. These results suggest that proteasome activity is involved in the early stages of viral infection. We next treated the IBV-infected cells with these inhibitors at 0–6 h.p.i. and harvested the samples at 12 h.p.i. to evaluate the level of positive and negative sense viral RNA. The generation of the negative-stranded genome represented the replication of the genome of the virus. Results showed that the levels of positive and negative sense viral RNA were greatly reduced in both cell types (Figure 2C), suggesting the virus genome replication is inhibited by these inhibitory treatments. By using the TCID_50_ assay, it was found that the release of progeny viral particles was significantly decreased to the minimum level by MG132 treatment at 0–6 h.p.i. but not by 6–12 h.p.i. treatment (Figure 2D). These data reveal that proteasome activity is required during the early stages of infection.

**Figure 3:**
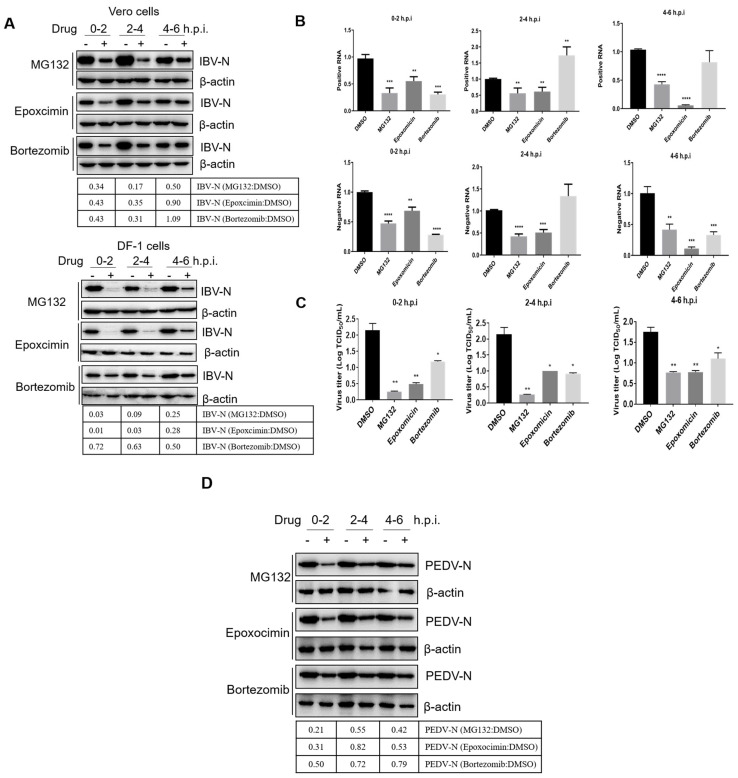
The active proteasome is required for IBV invasion at 0–4 h.p.i. (**A**) Vero or DF-1 cells were infected with 1 MOI of IBV and incubated with proteasome inhibitors at 0–2 h.p.i., 2–4 h.p.i., and 4–6 h.p.i. Cells were collected at 8 h p.i. to detect IBV N protein by using western blot. (**B**) Vero cells were infected with 1 MOI of IBV and incubated with proteasome inhibitors at 0–2 h.p.i., 2–4 h.p.i., and 4–6 h.p.i. Cells were collected at 8 h.p.i. for detection of positive and negative sense viral RNA levels by using RT-qPCR. (**C**) The cell culture medium in (**B**) was collected at 8 h.p.i. for detection of virus yield by using TCID_50_. The experiments in B and C were performed in triplicate, and the average values with stand errors were calculated and presented in a bar graph panel. (**D**) Vero were infected with 1 MOI of PEDV and incubated with proteasome inhibitors at 0–2 h.p.i., 2–4 h.p.i., and 4–6 h.p.i. Cells were collected at 8 h p.i. to detect the expression of PEDV N protein by western blot. The intensity of the IBV-N or PEDV-N band was determined with image J, normalized to β-actin, and shown as a fold change of MG132:DMSO, Epoxomicin:DMSO, and Bortezomib:DMSO. The asterisk (*) represents the *p*-value of the statistical test. * *p*-value < 0.1; ** *p*-value < 0.05 (significant); *** *p*-value < 0.01 (very significant); **** *p*-value < 0.001 (highly significant).

The early infection events, including attachment, endocytosis, intracellular transport, membrane fusion, and uncoating, occur approximately at 0–4 h.p.i. [14], while initial protein translation (the synthesis of gene 1 encoded replicases), virus genome replication, and viral structural and accessory protein translation are initiated at about 4–6 h.p.i. [76,77]. To further dissect the IBV infection steps controlled by UPS, we subdivided the inhibitor treatment time course according to virus invasion steps: 0–2 h.p.i., 2–4 h.p.i., and 4–6 h.p.i., and collected cells at 8 h.p.i. to detect the viral protein expression, genome replication, and progeny virus release. As shown in Figure 3A, in both Vero and DF-1 cells, the synthesis of IBV N protein was greatly decreased by all three proteasome inhibitor treatments at 0–2 h.p.i. and 2–4 h.p.i. but was only moderately suppressed by treatment from 4–6 h.p.i. in DF-1 cells, with even less suppression in Vero cells. The levels of positive and negative sense viral RNA were decreased for all three time periods by MG132 and Epoxomicin, while Bortezomib reduced RNA levels when present at 0–2 h.p.i. but not at later time points (except for 4–6 h.p.i. in DF-1 cells) (Figure 3B). Consistently, the release of progeny virus was also decreased by the treatment of proteasome inhibitors at 0–2, 2–4, and 4–6 h.p.i. The earlier treatment was applied, the more significant inhibition effect was observed (Figure 3C). With these data, we conclude that the timing when an active proteasome is needed for IBV infection is between 0–6 h.p.i., especially needed for invasion events at 0–4 h.p.i., the timing of attachment, endocytosis, cellular transport, membrane fusion, uncoating, and initial translation.

To further examine whether UPS plays a role in pan-coronavirus infection, we infected Vero cells with *α-coronavirus* PEDV and treated cells with different inhibitors at 0–2 h.p.i., 2–4 h.p.i., and 4–6 h.p.i. As the results show in Figure 3D, UPS inhibitors MG132 and Epoxomicin significantly inhibited PEDV infection at 0–2 h.p.i. and moderately suppressed PEDV infection at 2–4 h.p.i. and 4–6 h.p.i. Therefore, UPS is involved in coronavirus early infection events.

### 3.3. Proteasome Inhibitors Do Not Interfere with IBV Internalization

**Figure 4:**
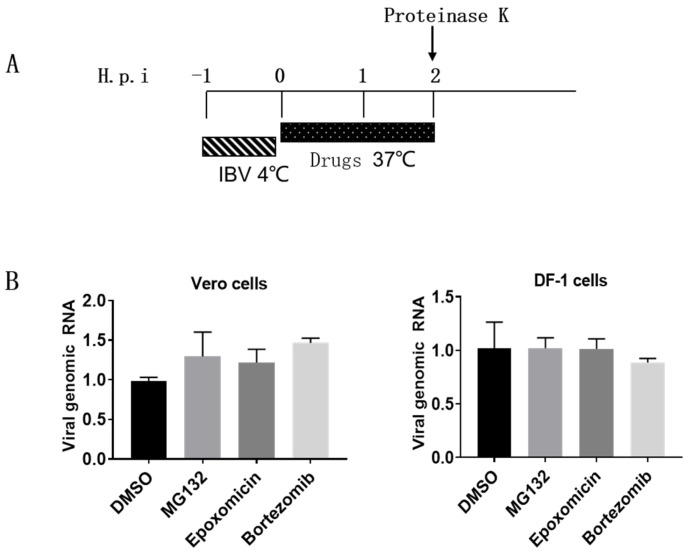
Proteasome inhibitors do not interfere with IBV internalization. (**A**) Experimental design showing the time of infection, drug treatment, and incubation temperature. (**B**) Vero or DF-1 cells were incubated with IBV (MOI = 1) at 4 °C for 1 h. The unbound virions were washed away with PBS, and the cells were incubated at 37 °C in the presence of proteasome inhibitors. At 2 h.p.i., cells were treated with proteinase K to remove the cell surface virus and subjected to RT-qPCR to measure the incoming virus genomic RNA. The experiment was performed in triplicate, and the average values with stand errors were calculated and presented in a bar graph panel.

Next, we attempted to dissect which virus invasion step UPR participates in [43,51]. Firstly, we examined whether proteasome inhibitors suppress IBV internalization. Vero cells and DF-1 cells were incubated with IBV at 4 °C for 1 h to allow viruses to attach and bind to the cell surface, then the medium was removed and replaced with fresh culture medium and incubated at 37 °C for 2 h, allowing virus endocytosis and internalization in the absence or presence of inhibitors [52]. After that, the cells were treated with proteinase K to remove the cell surface virus, and cells were harvested for measurement of internalized viral genomic RNA by RT-qPCR. As shown in Figure 4B, compared with the DMSO-treated group, proteasome inhibitors exerted no effect on the level of the incoming virus genome, suggesting that an active proteasome is not needed for IBV internalization.

### 3.4. Proteasome Inhibitors Interfere with IBV and PEDV Membrane Fusion

In our previous study, we showed that IBV entry mainly depends on clathrin-mediated endocytosis [14]. By using the R18/DiOC-labeled virus, we observed that virus particles moved along with the classical endosome/lysosome track, and membrane fusion was induced after 1 h p.i. in the late endosome/lysosome [14]. Here, we investigate whether the UPS is required for IBV intracellular endosome/lysosome trafficking or membrane fusion with endosome/lysosome. To make the incoming virus particles visible under a confocal microscope, R18-labeled IBV was applied to Vero cells in the presence of DMSO or 10 μM MG132. Specific antibodies were used to detect Rab5 (early endosome marker), Rab7 (late endosome marker), and LAMP1 (lysosome marker) at 1 and 2 h.p.i. As shown in Supplementary Figure S1, R18-IBV (red signal) mainly co-localized with Rab5 (yellow dots) at 1 h.p.i., while at 2 h.p.i., more R18-IBV particles accumulated in the late endosome or lysosome, which is marked by colocalization with Rab7 or LAMP1 (yellow dots). There was no much R18-IBV signal difference between the DMSO-treated and MG132-treated groups at both 1 and 2 h.p.i., suggesting the UPS is not involved in the IBV early-late endosome trafficking.

**Figure 5:**
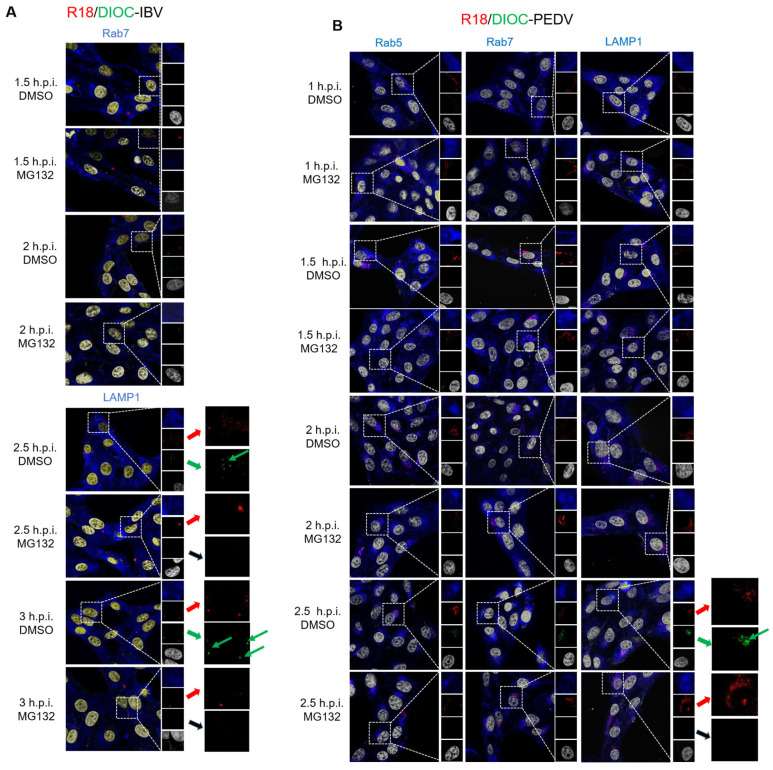
Proteasome inhibitors interfere with IBV and PEDV membrane fusion with late endosomes or lysosomes. (**A**,**B**) Vero cells were infected with 5 MOI of R18/DiOC-labeled IBV or R18/DiOC-labeled PEDV in the presence of DMSO or MG132. Cells were subjected to immunofluorescence with anti-Rab5, anti-Rab7, or anti-LAMP1 antibodies at the indicated time points. The separation of R18 (red) and DiOC (green) signals and the late endosomal or lysosomal marker (blue) were observed under an LSM880 confocal laser-scanning microscope. Representative images were shown. Red signals and red arrows represent the R18-labeled virus; green signals and green arrows represent the membrane fusion released by DiOC; blue signals represent early endosomes (Rab5), later endosomes (Rab7), and lysosomes (LAMP1).

To further examine whether UPS is required for the membrane fusion between virus and intracellular vesicle membranes, IBV was labeled with two fluorescent lipids, R18 (red) and DiOC (green), a method developed by Sakai et al. [78]. Here, high concentration of R18 (6.7 mM) was applied to quench the green fluorescence emitted by the DiOC. The intact virus membrane will display the red color of R18. When membrane fusion happens, the two lipids are diluted, and the green signal of DiOC is no longer quenched by R18. The red and green signals will be displayed, respectively. We have used this method to demonstrate that IBV fused with late endosomes or lysosomes at 2 h.p.i. in a previous study [14]. Vero cells were infected with R18/DiOC dual-labeled IBV in the presence of 10 μM MG132 or an equal volume of DMSO for 1 h, 1.5 h, 2 h, and 3 h, then subjected to Rab 7 or LAMP1 staining and observed under a confocal microscope. As shown in Figure 5A, at 1 and 1.5 h.p.i., there were only red dots observed; at 2 and 3 h.p.i., the DMSO-treated cells displayed the red and green dots signals (indicated with green arrows), suggesting membrane fusion happens from 2 to 3 h.p.i.; however, in the MG132-treated cells, only red dots signals were observed and there were no green dots, suggesting that the fusion of the virus membrane with endosomal or lysosomal membranes is blocked. These results reveal that the MG132 treatment prevents the IBV-endosomes/lysosomes membrane fusion but does not inhibit the internalization of virus particles.

To further confirm that UPS is involved in the membrane fusion step in pan-coronavirus infection, we labeled *α-coronavirus* PEDV with R18 and DiOC and applied the labeled virus onto Vero cells in the presence of 10 μM MG132 or an equal volume of DMSO. Cells were subjected to Rab5, Rab7, or LAMP1 staining at 1, 1.5, 2, and 2.5 h.p.i. As shown in Figure 5B, there were only red dots signals observed from 1 to 2 h.p.i., and green dots appeared at 2.5 h.p.i. in the DMSO-treated cells (indicated with green arrows), indicating that PEDV fuses with the endosomal of lysosomal membranes at 2.5 h.p.i.; interestingly, in the MG132-treated cells, there were only red dots signals but no green dots signals observed, indicating that MG132 blocks the membrane fusion process. These results further confirm that the UPS is involved in the pan-coronavirus membrane fusion invasion step.

### 3.5. Proteasome Inhibitors Interfere with IBV and PEDV N Protein Degradation and Uncoating

**Figure 6:**
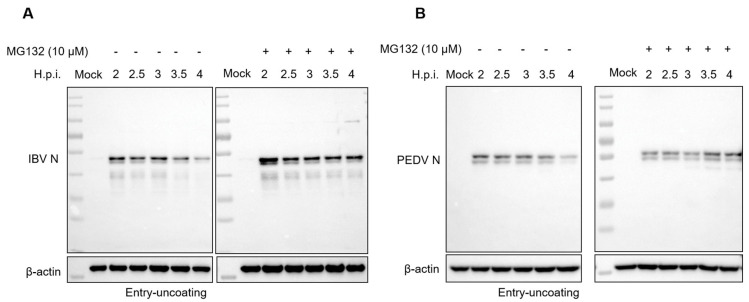
MG132 prevents the degradation of the incoming N protein during IBV and PEDV infections. (**A**,**B**) Vero cells were infected with 10 MOI of IBV (**A**) or PEDV (**B**) in the presence or absence of MG132 and subjected to western blot for detection of the incoming N protein level at 2, 2.5, 3, 3.5, and 4 h.p.i.

After membrane fusion, the coronavirus nucleocapsid is released into the cytosol by uncoating, and the RNA genome then serves as a template for polyprotein 1a and 1ab translation. As UPS is required for membrane fusion, we speculate that the inhibition of UPS will affect the following: uncoating and genomic RNA release. It has been reported that SARS-CoV-2 and PEDV N proteins are ubiquitinated and degraded in cells [79,80]. Whether the incoming N protein within the virion is degraded and involved in the release of genomic RNA is unclear. Here, we infected Vero cells with 10 MOI of IBV or PEDV, respectively, in the presence of 10 μM MG132 or an equal volume of DMSO, and harvested the cells at 2, 2.5, 3, 3.5, and 4 h.p.i. Western blot results in Figure 6A,B showed that along with the infection time course (2–4 h.p.i.), the incoming IBV N and PEDV N protein signal decreased, especially at 3.5 h.p.i. and 4 h.p.i., suggesting both IBV and PEDV N proteins are degraded after membrane fusion. The MG132 treatment recovered the N protein level, indicating the prevention of the degradation of N protein.

### 3.6. Proteasome Inhibitors Interfere with IBV Initial Translation

**Figure 7:**
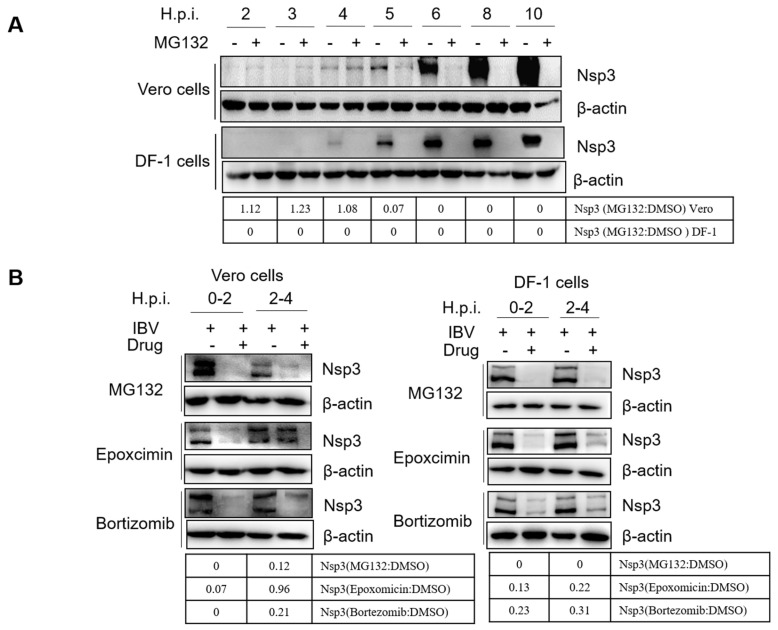
Proteasome inhibitors interfere with IBV initial translation. (**A**) Vero and DF-1 cells were infected with 1 MOI of IBV, treated with MG132 or DMSO, and subjected to Western blot for detection of the expression of the initial translation product nsp3 at 2, 3, 4, 5, 6, 8, and 10 h.p.i. (**B**) Vero and DF-1 cells were infected with 1 MOI of IBV and treated with proteasome inhibitors at 0–2 and 2–4 h.p.i. Cells were harvested at 5 h p.i. and subjected to western blotting for detection of the expression of nsp3. The intensity of IBV nsp3 was determined with image J, normalized to β-actin, and shown as a fold change of MG132:DMSO, Epoxomicin:DMSO, and Bortezomib:DMSO.

Next, we measured the initial translation of the virus genome. The initial translation employs the incoming virus genome as an RNA template and produces the replicases required for genome replication/transcription. Thus, initial translation occurs before the replication of the virus genome [81]. Nsp3 is produced by initial translation from gene 1. Here, we measured the expression of nsp3 by western blot to monitor the occurrence of initial translation [82]. Firstly, we examined the time point of the initial translation. Cells were infected with IBV, treated with MG132 or DMSO, harvested at 2, 3, 4, 5, 6, 8, 10 h.p.i., and subjected to western blot by using a polyclonal nsp3 antibody. Results in Figure 7A showed that the nsp3 was detected as early as 4 h.p.i., and the signal was increased along the infection time course, revealing the initial translation occurred at approximately 4 h.p.i. The MG132 treatment greatly suppressed the synthesis of nsp3 in both Vero and DF-1 cells, indicating that UPS affects initial translation. As the signal of nsp3 is weak at 4 h.p.i., we harvested cells at 5 h.p.i. in the following experiment to further clarify whether the proteasome inhibitors interfere with initial translation. Vero and DF-1 cells were incubated with IBV and treated with proteasome inhibitors at 0–2 and 2–4 h.p.i. and were harvested at 5 h.p.i. Western blot results showed that nsp3 synthesis was greatly suppressed by proteasome inhibitors at both 0–2 and 2–4 h.p.i. in both Vero cells and DF-1 cells, with the exception of epoxocimin at 2–4 h.p.i. in Vero cells (Figure 7B). Thus, the active proteasome is probably not only involved in membrane fusion and uncoating to release genomic RNA but also in the initial translation of the genomic RNA. It is worthy to note that the inhibition of endosomal escape or uncoating will also result in the inhibition of subsequent viral RNA translation.

### 3.7. Ubiquitination of Cellular or Viral Proteins Is Necessary for IBV Entry

**Figure 8:**
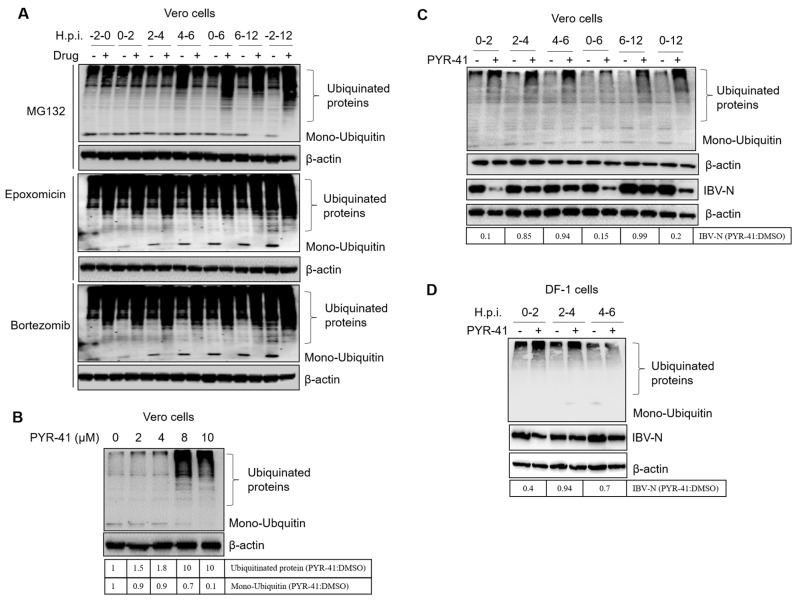
Ubiquitination of cellular or viral proteins is necessary for IBV entry. (**A**) Vero cells were treated with three proteasome inhibitors at −2–0, 0–2, 2–4, 4–6, 0–6, 6–12, and −2–12 h.p.i. and subjected to western blot at 12 h.p.i. by using a ubiquitin antibody. (**B**) Vero cells were treated with increasing concentrations of PYR-41 and subjected to western blot for detection of ubiquitinated proteins at 12 h post-treatment. (**C**) Vero cells were infected with 1 MOI of IBV and treated with 10 μM PYR-41 at 0–2, 2–4, 4–6, 0–6, 6–12, and −2–12 h.p.i. Cells were subjected to western blot for detection of ubiquitinated proteins and IBV N at 12 h.p.i. (**D**) DF-1 cells were infected with IBV and treated with 10 μM PYR-41 at 0–2, 2–4, 4–6 h.p.i. Cells were subjected to western blot for detection of ubiquitinated proteins and IBV N at 8 h.p.i. The intensity of IBV-N, ubiquitinated proteins, or mono-Ubiquitin was determined with image J, normalized to β-actin, and shown as a fold change of PYR-41:DMSO.

To determine whether the proteasome inhibitors interfere with protein degradation or protein ubiquitination, we measured the levels of ubiquitinated proteins and the abundance of the ubiquitin monomer. Cells were treated with proteasome inhibitors at −2–0, 0–2, 2–4, 4–6, 0–6, 6–12, and −2–12 h.p.i. and subjected to western blot at 12 h.p.i. by using a ubiquitin antibody. Results in Figure 8A showed that proteasome inhibition resulted in the accumulation of ubiquitinated proteins and the depletion of the ubiquitin monomer, especially by Epoxomicin or Bortezomib treatment. The inhibition of ubiquitinated protein degradation might prevent the recycling of ubiquitin, resulting in the depletion of the ubiquitin monomer. This will interfere with the new ubiquitination of cellular proteins or the incoming viral proteins. Based on the above experiment, we speculate that UPS inhibitors may interfere with the ubiquitination of proteins that are involved in virus invasion.

We next treated cells with PYR-41, a ubiquitin-activating enzyme E1 inhibitor, to suppress the ubiquitination process of cellular proteins [83]. Firstly, we determined the working concentration of PYR-41. Results showed that 8–10 μM PYR-41 led to the accumulation of ubiquitinated cellular proteins and decreased the free ubiquitin monomer (Figure 8B). Vero cells were then infected with IBV and treated with 10 μM PYR-41 at 0–2, 2–4, 4–6, 0–6, 6–12, and −2–12 h.p.i., and subjected to western blot at 12 h.p.i. Again, we observed the accumulation of ubiquitinated proteins and the depletion of ubiquitin monomers by PYR-41 treatment. It has been reported that in the presence of PYR-41, the ubiquitin monomer is in inactivated status, and downstream ubiquitination and ubiquitination-dependent protein degradation or other ubiquitination-mediated cellular activities are blocked [83]. Interestingly, in this study, we observed that the ubiquitinated proteins also accumulated in the presence of PYR-41, similar to proteasome inhibitor treatment. Inhibition of the ubiquitination process by PYR-41 at 0–2, 0–6, and 0–12 h.p.i. greatly decreased the infection by IBV, while inhibition of the ubiquitination process 2–4, 4–6, and 6–12 h.p.i. displayed less suppression on IBV infection (Figure 8C). Thus, PYR-41 mainly interferes with IBV infection at 0–2 h.p.i. In DF-1 cells, similar results were observed (Figure 8D). The above evidence demonstrates that the ubiquitination state of proteins in general also plays an important role during the IBV entry step.

## 4. Discussion

**Figure 9:**
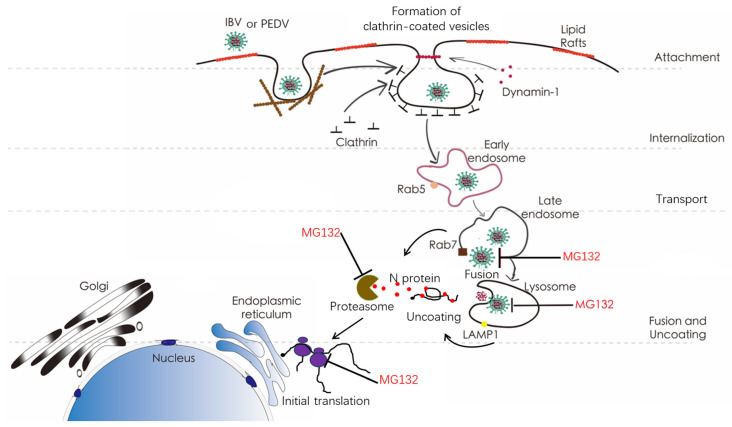
Cartoon representing the steps of the virus life cycle that may be susceptible to proteasome or ubiquitination inhibition.

IBV and PEDV are the two major coronaviruses posing a serious threat to poultry and porcine farms, respectively. To investigate the involvement of USP in the coronavirus life cycle, several proteasome inhibitors, including MG132, Epoxomicin, and Bortezomib, were applied at various coronavirus-specific time points across the life cycle, and the IBV or PEDV infection was measured. It was shown that proteasome inhibition resulted in the accumulation of ubiquitinated proteins while decreasing the availability of free cellular mono-ubiquitin. Thus, the decrease in virus infection could be explained as proteasome- or ubiquitin-dependent. We found that the UPS activity is required for virus infection when the proteasome inhibitors were added between 0–2 h.p.i. and 2–4 h.p.i., suggesting the UPS is required for early infection stages. By dual labeling IBV or PEDV virus particles by R18 and DiOC, it was found that addition of MG132 blocked the virus-cellular membrane fusion with the late endosomes or lysosomes, indicating that the UPS is required for endosomal or lysosomal escape of IBV and PEDV. This is consistent with the previous finding that the presence of MG132 makes the entering murine coronavirus accumulate in both the endosomes and lysosomes [52]. Based on the above evidence, we concluded that UPS is required for pan-coronavirus entry, especially at the membrane fusion step (Figure 9). We further treated cells with PYR-41, an E1 ubiquitin-activating inhibitor, to interfere with the ubiquitination modification of cellular or viral proteins. Results showed that ubiquitination inhibition significantly blocked IBV invasion at 0–2 h.p.i., further confirming that ubiquitination is required for virus entry events.

The detailed molecular mechanisms for coronavirus virus entry involve the binding of virus particle surface S protein to the host cell receptor and fusion at the plasma membrane or after being trafficked to late endosomes or lysosomes under low pH conditions. A crucial event in the membrane fusion process is the proteolytic cleavage of the viral spike protein by the host proteases that releases the fusion peptide, enabling fusion with the host membrane system. Multiple host proteases, such as furin, transmembrane protease serine 2 (TMPRSS2), and cathepsins, cause the S protein to become fusion-competent. Our previous study showed that IBV particles are transported into endosomes and then into lysosomes during the early stages of infection, which are mediated by clathrin-mediated endocytosis [14]. Endocytic pathways play an important role for pan-coronaviruses to penetrate the cell membrane barrier, such as PEDV [15,16], SARS-CoV-2 [84,85], HCoV-NL63 [86], HCoV-229E [87], SARS-CoV and MERS-CoV [88], and MHV [52]. Membrane fusion in late endosomes or lysosomes usually requires low pH-dependent proteases to cleave the S protein and expose the N-terminal fusion peptide of S2 to host membranes. Thus, successful membrane fusion requires the cleavage of S proteins, the insertion of fusion peptides into the cellular membrane, and the conformation change of S2 proteins. All these events are critical steps for virus entry and become potential targets for antiviral drug development. For example, the endogenous proteases responsible for S protein cleavage are usually targeted by inhibitor design [89], while the S2 fusion core (six-helix bundle formation) is also targeted by small molecules of polypeptide drugs [90]. In this study, we found that proteosome inhibitors inhibit the coronavirus membrane fusion with late endosomes or lysosomes, suggesting these inhibitors are attractive candidates for developing antivirals to control coronavirus infection. It is not clear whether UPS is required for the successful cleavage of S protein or the following conformation change of the S2 subunit and successful membrane fusion.

By examining the incoming N protein level at 0–4 h.p.i., we found that both IBV and PEDV N protein decreased from 3.5–4 h.p.i., the time following escape of the genome and associated proteins from late endosomes or lysosomes, and the application of MG132 recovered the incoming N protein level. The possible underlying mechanisms for this phenomenon are: (1) the IBV and PEDV virus disassembly and uncoating are dependent on the N protein degradation to release genomic RNA for initial translation, and the N protein degradation depends on proteasome activity; MG132 blocks the proteasome activity and prevents the N protein degradation, thereby interfering with the uncoating events; (2) The treatment with MG132 has already prevented virus-endosomes/lysosomes membrane fusion, resulting in the arresting of the virus particles in the late endosome; the N protein is associated with genomic RNA inside the virion and remains within the late endosomes, thereby avoiding proteasomal degradation in the cytosol. The ubiquitination-mediated degradation (proteasomal or autophagic pathway) of coronavirus N protein has already been reported, which is speculated to be a host anti-viral response by impairing virus assembly [79,80,91,92,93]. Here, we found that the incoming N protein is degraded at the time point consistent with uncoating. This degradation may help release genomic RNA as a template for the subsequent initial translation of viral replicases. Similar to our finding, it is reported that dengue virus genome uncoating requires viral capsid protein ubiquitination and degradation [50]; the core of poxvirus vaccinia virus (VACV) is involved in proteasome-dependent degradation to release the viral DNA for replication [43,94,95]; and African swine fever virus (ASFV) uncoating involves de-capsidation of the virion core mediated by proteasome-dependent degradation of the proteinaceous core shell surrounding the DNA [96]. Thus, the involvement of UPS in virus uncoating is common in various virus families.

After uncoating, coronavirus genomic RNA separates from N protein to initiate the synthesis of polyproteins 1a and 1ab, which are then cleaved into 15–16 nsps involved in the replication/transcription of virus genomic RNA and subgenomic mRNA. We examined whether proteasome inhibitor MG132 suppresses the gene 1 initial translation by detecting the expression of gene 1 encoded nsp3, which is one of the initial translation products and is not present in the incoming virus particle. We found that nsp3 was detected at 4 h.p.i. and increased along the infection time course; the presence of MG132 inhibits the initial expression of nsp3. The suppression of initial translation might be due to the blockage of membrane fusion or uncoating by MG132 treatment; however, it cannot be excluded that MG132 might directly target the initial translation step.

## 5. Conclusions

Taken together, our results provide new information that the proper function of UPS is required for coronavirus-host membrane fusion and the degradation of incoming N protein at the post-fusion step for genome uncoating. The underlying mechanisms of proteasome inhibitors’ effect on coronavirus membrane fusion, uncoating, or initial translation require further investigation. Hopefully, a better understanding of the coronavirus-host interaction and the virus processes at the molecular level will provide the necessary tools for coronavirus control.

## Data Availability

The data presented in this study are available in this article and Supplementary Materials.

## Supplementary Materials

The following supporting information can be downloaded at: https://www.mdpi.com/article/10.3390/v15102001/s1, Figure S1: Vero cells were infected with 5 MOI of R18-labeled IBV and incubated 410 with DMSO or MG132.
