# Supplementary material for: The Ubiquitin-Proteasome System Facilitates Membrane Fusion and Uncoating during Coronavirus Entry
Source: Viruses. 2023 Sep 26;15(10):2001. doi: 10.3390/v15102001 (PMC10610886; doi:10.3390/v15102001)
Supplement: Supplementary file 1 [file viruses-15-02001-s001.zip › viruses-2589936-SI.pdf]

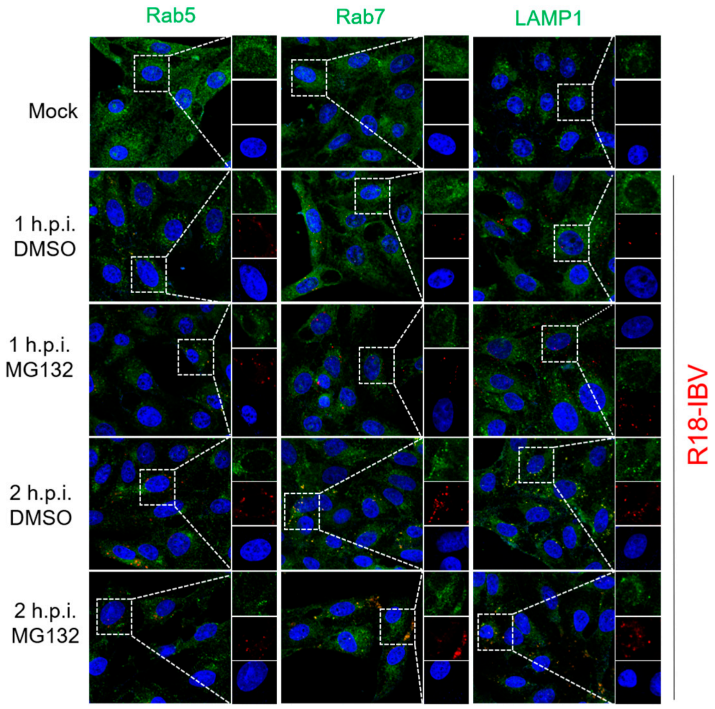

Supplementary Figure S1. Vero cells were infected with 5 MOI of R18-labeled IBV and incubated with DMSO or MG132. Cells were subjected to immunofluorescence with anti-Rab5, anti-Rab7, or anti-LAMP1 antibody at 1 or 2 h.p.i., and nuclei were stained with DAPI. The signals were observed under LSM880 confocal laser-scanning microscope (Zeiss). Red signal represents R18-IBV; green signals represent early endosomes (Rab5), later endosomes (Rab7), and lysosomes (LAMP1); blue signals represent nuclei.
